# Hierarchical viscosity of aqueous solution of tilapia scale collagen investigated via dielectric spectroscopy between 500 MHz and 2.5 THz

**DOI:** 10.1038/srep45398

**Published:** 2017-03-27

**Authors:** H. Kawamata, S. Kuwaki, T. Mishina, T. Ikoma, J. Tanaka, R. Nozaki

**Affiliations:** 1 Department of Physics, Faculty of Science, Hokkaido University, Sapporo 060-0810, Japan; 2 Department of Metallurgy and Ceramics Science, Tokyo Institute of Technology, Tokyo 152-8550, Japan

## Abstract

Aqueous solutions of biomolecules such as proteins are very important model systems for understanding the functions of biomolecules in actual life processes because interactions between biomolecules and the surrounding water molecules are considered to be important determinants of biomolecules’ functions. Globule proteins have been extensively studied via dielectric spectroscopy; the results indicate three relaxation processes originating from fluctuations in the protein molecule, the bound water and the bulk water. However, the characteristics of aqueous solutions of collagens have rarely been investigated. In this work, based on broadband dielectric measurements between 500 MHz and 2.5 THz, we demonstrate that the high viscosity of a collagen aqueous solution is due to the network structure being constructed of rod-like collagen molecules surrounding free water molecules and that the water molecules are not responsible for the viscosity. We determine that the macroscopic viscosity is related to the mean lifetime of the collagen-collagen interactions supporting the networks and that the local viscosity of the water surrounded by the networks is governed by the viscosity of free water as in the bulk. This hierarchical structure in the dynamics of the aqueous solution of biomolecules has been revealed for the first time.

Aqueous solutions of biomolecules such as proteins are very important model systems for understanding the functions of biomolecules in actual life processes because interactions between biomolecules and water are considered to be an important determinant of biomolecules’ functions^1^,^2^. Dielectric spectroscopy is one of the powerful tools used to investigate aqueous solutions of biomolecules, especially from the viewpoint of molecular dynamics^3^,^4^,^5^,^6^,^7^,^8^,^9^,^10^,^11^,^12^,^13^. Usually, mixing of water and biomolecules provides a variety of molecular dynamics in the solution, depending on the type of biomolecules. Therefore, in the case of an experimental study via dielectric spectroscopy of a solution, we should pay attention to the dynamics of solute molecules and water molecules and interactions between them due to mixing. It is commonly understood that protein molecules form specific conformations in water suspension and the biological function enters via bound waters surrounding the surface of the proteins^2^. Thus far, aqueous solutions of globule proteins have been extensively studied by many researchers via dielectric spectroscopy. Three relaxation processes are commonly observed in aqueous solutions of globule proteins such as myoglobin in the frequency range from 1 MHz to 20 GHz^7^,^8^,^9^. The low-frequency process observed at approximately 1 MHz has been attributed to rotational diffusion of the protein molecule, whereas the high-frequency process at approximately 20 GHz is considered to be due to the bulk water. In contrast, the intermediate process at approximately 10–100 MHz has smaller dielectric relaxation strength compared with the other two processes. One of the possible ideas for understanding the intermediate process is bound water. The bound water has strong interactions with the surface of the protein molecule and such water molecules have restricted mobility. However, the origin of the intermediate process is still not very clear.

Collagens are proteins mainly supporting biological structures such as bone and skin^1^,^2^. A collagen molecule is composed of three linear peptide chains (α chains) with a triple-helix structure. A complete triple-helix collagen molecule is assembled with the assistance of propeptides in the life process, whereas the linear peptide chains form only subsequent molecular complexes with an incomplete triple-helix interaction in an aqueous solution. Because the process of self-assembly of collagen molecules into fibril is considered to be very useful in regenerative medicine, application studies have been extensively performed, especially in the medical field. Recently, attention has been focused on collagen molecules extracted from fish because this avoids risks of zoonosis^14^.

Usually, natural collagens extracted from living material are not easily soluble in water. Therefore, to the best of our knowledge, systematic investigations of collagen aqueous solutions have not been performed thus far. In contrast, purified collagen, so-called “atelocollagen”, which is obtained under chemical treatments with pepsin, is water-soluble. Ikoma *et al*. have established a technical method to extract atelocollagen molecules from the scales of tilapia (*Oreochromis niloticas*)^14^. Moreover, the denaturation temperature of this collagen is in the range between 301 and 316 K. These properties make it possible to provide purified collagen molecules and perform fundamental experimental studies without paying special attention to the handling temperature.

Because fluids of collagen-water systems have a viscous nature^15^, the relation between the viscosity and the water bound to the collagen molecules has often been discussed in the same manner as the solution dynamics of globule proteins such as myoglobin^7^,^8^,^9^,^10^. Note that a certain amount of water molecules bound to the solute molecules is required to provide a high viscosity in the aqueous solutions of such globule proteins. However, the fundamental details of the causes of this high viscosity have not been examined for the collagen solutions thus far.

In this study, we investigated characteristic features of molecular dynamics in a collagen aqueous solution via dielectric spectroscopy for the first time. It was found that there is no difference between dielectric dispersions of the viscous collagen aqueous solutions and pure water; even the value of viscosity of the solution is one-hundred-times greater than that of pure water. We also studied a solution with amino acids to evaluate the microscopic viscosity. Aqueous solutions of amino acids exhibit a dielectric relaxation process at approximately 100 M-1 GHz and that the value of relaxation time can be well described by the extended Stokes-Einstein-Debye law, employing the viscosity of bulk water^11^,^12^. It was found that relaxation frequencies of the process of amino acids for both the aqueous solution of collagen and pure water are the same. These experimental results suggest that the interaction between water and collagen molecules is not very strong and that most of the water in the solution is free, as in bulk pure water. It can be concluded that the collagen molecules form a network structure surrounding the water molecules and the mean lifetime of the collagen-collagen interactions is responsible for the high viscosity of the collagen aqueous solution. The macroscopic viscosity is different from the microscopic local viscosity. This hierarchical structure in the dynamics of an aqueous solution of biomolecules has been revealed for the first time.

## Results and Discussion

### Viscosity and dielectric dispersion of collagen aqueous solution

**Figure 1:**
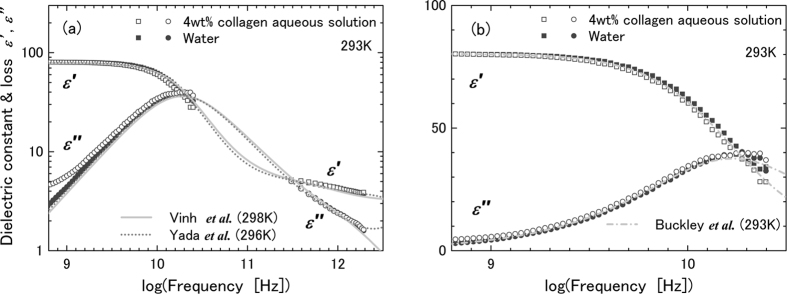
Dielectric constant and loss of collagen aqueous solution and pure water. Dielectric constant and loss of 4 wt% collagen aqueous solution (open symbols) and pure water (filled symbols) in the frequency range of (**a**) TDR and THz-TDS and (**b**) TDR measurements. In Fig. 1(a), experimental results reported in the literature^32^,^33^ are also shown for comparison. Our result for water agree with the reference data and other reports^34^,^35^, thus suggesting that our experimental setup works well in the measured frequency range. A surprising fact is that there is essentially no difference between the experimental values for the complex permittivity of pure water and that of the aqueous solution of collagen, although the viscosity values are very different. Similar results were obtained for all of the collagen aqueous solutions with different concentrations. This result can be examined in detail in Fig. 1(b) for the TDR frequency range. The values of the dielectric constant and loss for the pure water and the aqueous collagen solution behave in the same manner in the entire frequency range of the TDR measurements. Moreover, these are well described using the dielectric parameters of water at the same temperature reported in the literature^34^. Adding collagen to pure water does not greatly affect the dielectric dispersion of the water in the measured frequency range. Note that dielectric loss for the collagen aqueous solution at lower frequencies, approximately 1 GHz, is slightly greater than that for the pure water. It seems that this deviation is due to the large contribution of the dc conductivity of the solution caused by ionic contamination.

First, we prepared four samples of aqueous solution with different collagen concentrations. All the samples prepared were viscous liquids, and the viscosity of each sample increased with increasing collagen concentration. These sample conditions are listed in Table 1. Figure 1 shows experimental results regarding the complex permittivity of pure water and aqueous solution of collagen (4 wt%). Essentially no difference was found between the experimental values for the complex permittivity of pure water and those of the aqueous solutions of collagen, although the values of viscosity are quite different from each other. This is quite in contrast to the fact that adding globule proteins into water affects the dielectric relaxation process of water because of the decreased bulk water fraction^10^.

### Effect of the collagen volume fraction on static dielectric constant

Generally, the apparent value of the dielectric constant of solutions is proportional to the volume fraction of the polar component. In the case of aqueous solutions of collagen, increased collagen concentration decreases the volume fraction of water. The dielectric relaxation process caused by the fluctuation of an entire collagen molecule should be observed at much lower frequencies. Therefore, increasing the collagen concentration decreases the experimental value of the dielectric constant in the time-domain reflectometry (TDR) frequency range. We have estimated the volume fraction of collagen molecules in the solution. Assuming that the collagen molecule is a rod with a section diameter of 1.5 nm and length of 300 nm, the ratio of the volume fraction to the concentration is estimated to be 0.01 per wt%. This means that the decrease of the dielectric constant of the 4 wt% collagen solution from pure water is only 3.2. A decrease of 3.2 in the dielectric constant is very difficult to detect in our experimental setup using TDR measurements.

### Effect of the free water fraction on the static dielectric constant

Note also the amount of water molecules in the bulk. It has been reported that water-soluble globule proteins and polymers hold the bound water in their aqueous solutions. This effect reduces the amount of free water in the bulk. Crystal structures of model collagen systems with water have been extensively studied via X ray diffraction measurements and thermal analysis^1^,^16^,^17^. It has been suggested that the collagen triple-helix structure binds one or several water molecules, the so-called “bound water” per amino acid residue. It has also been reported that such bound water contributes to the stability of the triple-helix structure^17^. According to a simple estimation based on such reports for the bound water, the fraction of water molecules located on the collagen surface as the first layer with hydrophilic or hydrophobic interactions is only 1.5% at maximum. Again, the decrease in dielectric constant caused by this effect is very difficult to detect in our experimental setup.

### Behavior of amino acids in water

To understand the origin of the discrepancy in dielectric and viscosity behaviors, we introduced amino acids into the solutions to estimate the “local viscosity” of the solutions. It is well established that aqueous solutions of an amino acid exhibit a dielectric relaxation process on the lower-frequency side of the primary process of water^11^,^12^. The origin of the low-frequency dielectric relaxation process is considered to be rotational diffusion of an amino acid molecule. The relaxation time of the process can be explained using the Stokes-Einstein-Debye (SED) law with the viscosity of bulk water. Examining the behaviors of the low-frequency process due to an amino acid in pure water and aqueous collagen solutions is valuable for understanding the microscopic dynamics. We investigated the aqueous solutions in this manner with four amino acids (glycine, β-alanine, L-serine, and L-arginine).

**Figure 2:**
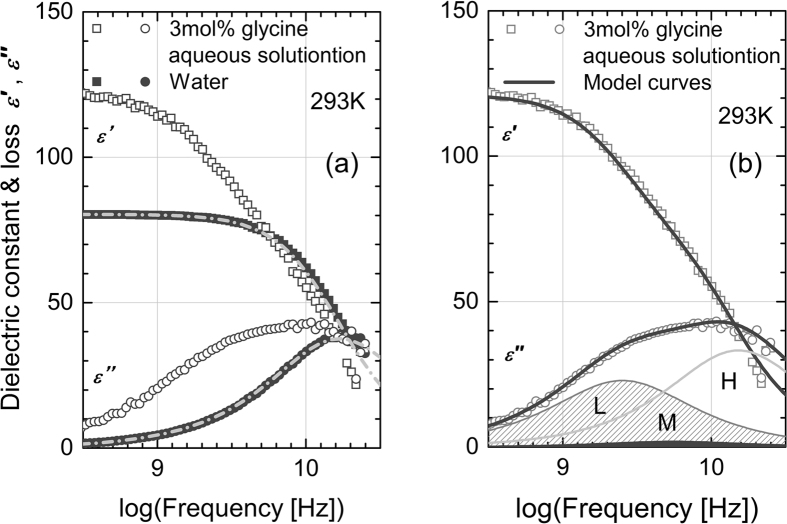
Dielectric constant and loss of glycine aqueous solution and pure water. Dielectric constant and loss of (**a**) 3 mol% glycine aqueous solution (open symbols) and water (filled symbols); (**b**) best-fitting curve for the aqueous solution of glycine. Figure 2(a) shows that the solution has a greater dielectric constant at lower frequencies and that an additional loss shoulder appears at the lower-frequency side of the water relaxation process due to adding glycine. Similar behaviors were observed in all the samples with different amino acids. These behaviors are consistent with those observed in previous works^11^,^12^. To analyze the complex permittivity in detail, we attempted to fit the experimental data using three Debye functions (Eq. 1). In previous papers, Sato *et al*.^11^ and Rodriguez-Arteche *et al*.^12^ introduced only two relaxation processes to describe their experimental results in a similar frequency range. However, they assumed one Debye function for the high-frequency process^11^,^12^ and one Cole-Cole function for the low-frequency process^12^. Combined with the aforementioned previous results, our results strongly suggest that the H process is due to bulk water and the L process is due to the rotational diffusion of the amino acid molecule. The H process is slightly affected by adding glycine, in contrast with the aqueous solution of collagen. Because an amino acid molecule is considered to behave as a zwitter ion under an appropriate pH, a significant amount of water corresponding to the amino acid molar fraction is taken from the bulk water owing to the hydration around the positive and negative charges. This effect decreases the value of the loss peak of the H process, as has been discussed in previous papers^11^,^12^. In contrast, the M process has not been mentioned in the previous dielectric studies^11^,^12^. Note that dielectric dispersion corresponding to the M process can be involved in the L process if we employ the Cole-Cole function for the L process. We do not further discuss the small M process in this paper.

As an example of experimental results, Fig. 2(a) shows the dielectric constant and loss of the aqueous solution of glycine (molar fraction *x* = 3%) and pure water at the frequencies of the TDR measurements. It was found that the dielectric constants and losses of all the aqueous solutions of amino acids could be well described using three Debye functions, as shown in Fig. 2(b). The function is


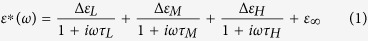


where ε^*^ = *ε*′ − *iε*′′ is the complex permittivity with the dielectric constant and loss, 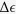 is the dielectric strength, τ is the dielectric relaxation time, *ε*_∞_ is the high-frequency limiting permittivity, and ω is an angular frequency. The subscripts L, M and H represent the low-, intermediate- and high-frequency processes, respectively. Combined with previous reports^11^,^12^, our results strongly suggest that the H process is due to the bulk water and the L process originates from rotational diffusion of the amino acid molecules. Here, we employed an extended SED law, the so-called Perrin’s equation^18^,^19^, to estimate the values of the dielectric relaxation time, *τ*_DR_, due to the rotational diffusion of the ellipsoidal molecule. The microscopic relaxation time, *τ*_rot_, with electric dipole moment parallel to the long axis is given by


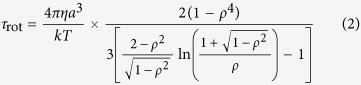


where 2*a* and 2*b* are lengths of the long and short axes, respectively. The axis ratio is defined as ρ = *b*/*a*. The dielectric relaxation time, *τ*_DR_, to be measured is related to *τ*_rot_ as follows:


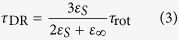


where *ε*_*S*_ is the static permittivity and *ε*_∞_ is the high-frequency limiting permittivity. In the present case, the values of *a* and *b* have been estimated from the molecular surface of amino acids using the Jmol software package^20^; 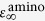 for the L process is used rather than *ε*_∞_. In this work, it was reconfirmed that the origin of the L process is rotational diffusion of the amino acid molecules, as indicated in Table 2(a).

### Behavior of amino acids in collagen aqueous solution

**Figure 3:**
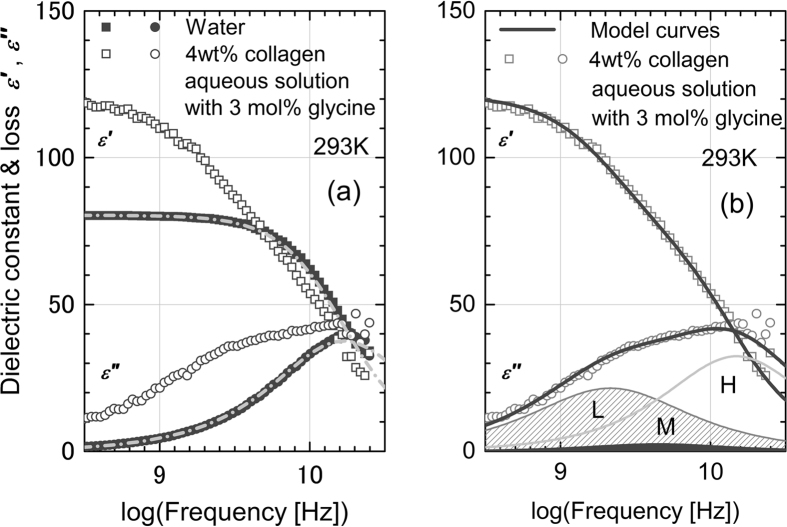
Dielectric constant and loss of collagen aqueous solution with glycine and pure water. Dielectric constant and loss of (**a**) 4 wt% collagen aqueous solution with 3 mol% glycine (open symbols) and water (filled symbols); (**b**) best-fitting curve for the solution using three Debye functions (Eq. 1). As evident for the aqueous amino acid solutions, all the sample solutions exhibit the L, M and H processes. The dielectric relaxation times of the L processes for all the samples are very similar to those of the corresponding aqueous solutions of amino acids, as indicated in Table 2(a) and (b). Combining the results shown in this figure and Table 2, it is strongly suggested that the local viscosity in the collagen aqueous solution is almost the same as that of bulk water.

**Figure 4:**
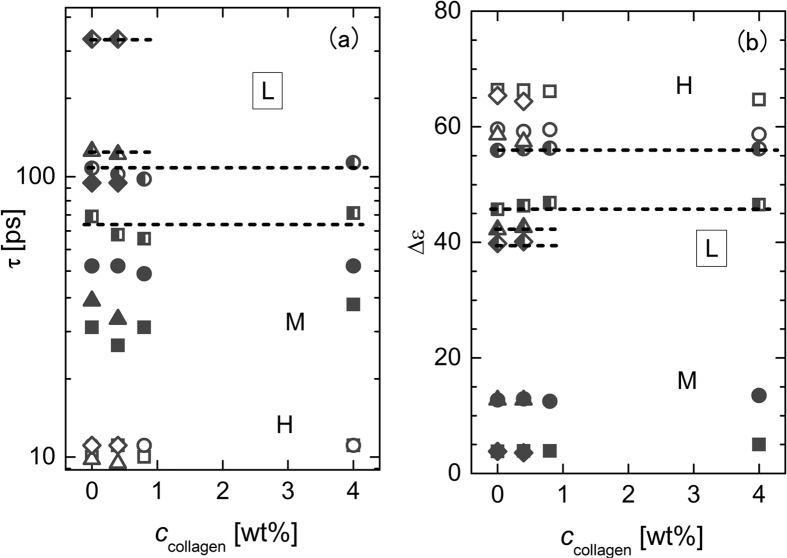
Relaxation times and strengths for the L, M, H processes versus collagen concentration. Plots of (**a**) dielectric relaxation time and (**b**) dielectric relaxation strength versus collagen concentration for dielectric relaxation process (half-filled symbol: L; filled symbol: M; open symbol: H) in aqueous collagen solutions with different amino acids (square: glycine; circle: β-alanine; triangle: L-serine; diamond: L-arginine). The values of the molar fractions of amino acids relative to water are listed in Table 2. In Fig. 4(a), the values of the relaxation time of the L, M, and H processes for all the amino acids employed in this study are plotted versus the concentration of collagen in the aqueous solutions. Figure 4(b) presents similar plots for the relaxation strength. At *c*_collagen_ = 0 (no collagen), the values for the relaxation time of the L process depend on the amino acid type, as discussed above. Note that the relaxation time and the strength of the L process for the specific amino acid are almost constant with collagen concentration within the experimental uncertainty.

Samples of aqueous solution of collagen with amino acids were prepared by adding collagen to the aqueous solution of an amino acid to avoid the difficulty of adding an amino acid to viscous aqueous collagen solutions. Figure 3(a) shows the complex permittivity of the aqueous solution of collagen with glycine. The frequency profiles of the complex permittivity shown in this figure are very similar to those of the aqueous solution of glycine shown in Fig. 2(a). Actually, the value of *τ*_*L*_ in the aqueous solution of glycine is almost the same as that in the aqueous collagen solution with glycine; such a similarity was found for all the amino acids, as indicated in Table 2. This fact strongly suggests that the local viscosity of the aqueous solution of collagen is not affected by the existence of the collagen molecules, even though the macroscopic viscosity increases drastically by adding collagen to water. To ensure that this consideration of the local viscosity is appropriate for the present collagen solutions, we studied the behavior of the L process in different concentrations of collagen. As shown in Fig. 4(a) and (b), the relaxation time and the strength for the L process with a specific amino acid are almost independent of the collagen concentration, *c*_collagen_. This result indicates that the local viscosity of the aqueous solution of collagen is independent of the macroscopic viscosity of the aqueous solution of collagen, even in the case of gel-like solutions, and there is essentially no interaction between the amino acid and the collagen molecules. This hierarchical viscosity in the aqueous solution of biomaterials has been observed for the first time in this work.

### Considerations regarding collagen aqueous solutions

Here, we consider the collagen aqueous solution in detail based on the experimental results described above. It seems possible that the increased viscosity in the aqueous solution of collagen is due to the development of types of network structures surrounding water constructed by rod-like collagen molecules. Most of the water remains as free water in the networks, even in the 4 wt% aqueous solution of collagen. This is not an unreasonable idea because a dielectric study of glassy hydrated collagen (~80 wt%) suggested that water has significant mobility even in a rigid collagen matrix below the denaturation temperature^21^. It is worth noting that the network for the aqueous solution of 4 wt% collagen molecules involves almost 2000 collagen rods in a cube with sides the length of the collagen (300 nm).

To form a network, collagen molecules should strongly interact with each other. Solutions of high molecular weight polymer exhibit high-viscosity or even gel-like characteristics^22^. In this case, the friction between flexible polymer chains with entanglement is important to provide such characteristics. However, this effect of interactions between collagen molecules would be very small in the case of collagen aqueous solutions because of the rod-like shape of collagen molecules and their lack of flexibility. In contrast, because the collagen molecule used in this work is “atelocollagen”, which does not possess N and C terminuses, both ends of the rod-like molecule have positive or negative charges in water, as for amino acids. Moreover, a certain amount of amino acids in the peptide chains composing the collagen molecule are acidic or basic. Therefore, there are certain amounts of possible positive or negative charges along the collagen molecule; these charges could cause a strong interaction. However, according to previous studies^11^,^12^ and the present work regarding aqueous solutions of amino acids, the electric interactions between charges along the solute molecules are not very strong in the aqueous solution. It has been suggested that the hydrated water molecules surrounding the charges mask the electric interactions between the charges^11^. It seems that such electric interactions are not responsible for forming collagen networks.

### Effect of adding HCl to collagen aqueous solution

To examine the interaction in the aqueous solution of collagen, we have studied a similar solution with a small amount of hydrochloric acid (HCl). Adding HCl to an aqueous solution of micro-fibrils helps to deform the micro-fibrils into collagen molecules^14^. We found that the value of viscosity of the 0.4% solution with HCl at pH 3 decreases drastically (from 113 to 68 [mPa · s]). This result indicates that HCl forces the interaction between collagen molecules to weaken. Note that adding HCl does not cause difference in the dielectric dispersions between the 0.4% aqueous solutions of collagen. The dielectric dispersions of aqueous HCl solutions with low HCl concentration, such as 0.001 mol/dm^3^ (pH3), at frequencies 200 M-25 GHz have been reported to be almost the same as that of pure water^23^. The HCl experiments in this work suggest that the interaction between collagen molecules in forming the network is related to the driving force of self-assembly of collagen molecules into micro-fibrils^14^,^24^,^25^. This interaction makes a connection between the collagen molecules with a characteristic lifetime in water. In this case, the viscosity of the collagen aqueous solution would be related to the mean lifetime of the interaction. Increasing the number density of collagen molecules in water results in more complicated connections, such as 3D networks. However, note that more accurate considerations for the network structure and dynamics must be made in further studies via X-ray or neutron scattering measurements.

### Applications to other systems

Our considerations regarding collagen aqueous solutions can be applied to other systems, such as hydrogels. Elastic and non-fluid characteristics of our aqueous solution of collagen with higher viscosity (>2 wt%) are similar to those of polymer hydrogels^26^. Polymer hydrogels consist of 3D polymer networks with water involved in the networks. The physical properties of polymer hydrogels have been investigated from many points of view. One of the curious properties of polymer hydrogels is their very unique mechanical characteristics, namely, their smoothness and softness without flow^26^. It is useful to consider the molecular dynamics of water in the polymer hydrogels from the microscopic point of view, as described in this work. Finally, we note that the results obtained in this work are also useful for understanding the life phenomena from the viewpoint of physics because living materials have essentially the same hierarchical structures in terms of their dynamics^2^.

## Methods

### Sample preparations

A sample of tilapia scale collagen (freeze-dried) was purchased from Taki Chemical [FD-08G]. This type I collagen is “atelocollagen”, which is a purified triple-helix rod-like molecule (1.5 nm *ϕ* × 300 nm, *M*_w_ = 300 k) that consists of three α chains with –NH_3_^+^ and –COO^−^ terminuses in water with natural pH. The collagen-gelatin denaturation temperature is approximately 301–316 K. The α peptide chain in the tilapia scale collagen molecule consists of approximately 1000 amino acids. The ratios of amino acid components in this collagen molecule have already been reported in the literature^14^. In this study, all the experimental processes, including sample storage and treatment, were performed at temperatures of less than 298 K. Glycine (>99%) was purchased from Kishida Chemical, β-alanine (>99%) and L-serine (>99%) were obtained from Tokyo Chemical Industry, and L-arginine (>98%) was purchased from Kanto Chemical. All the materials were used as received. Pure water was obtained using a water purification system [Millipore Milli-DI].

### Viscosity measurements

The viscosity values of the sample solutions were measured using a viscometer [Tuning Fork Vibro Viscometer, A&D SV-1A]. This viscometer enables measuring the viscosity and temperature of a fluid sample simultaneously.

### Dielectric measurements

Complex permittivity measurements were performed via TDR and terahertz time-domain spectroscopy (THz-TDS) to cover a wide frequency range between 500 MHz and 2.5 THz. For frequencies from 500 MHz to 25 GHz, the TDR system was employed. The basic instrumentation has already been reported^27^. In this study, we used a digital sampling oscilloscope [HP54750A] with a TDR plug-in [HP54753A]. The flat end of a 2 mm*ϕ* semi-rigid coaxial cable was used as a capacitor, thus forming electrodes for the dielectric measurements. The coaxial cable was composed of copper-nickel outer and inner conductors with a polytetrafluoroethylene (PTFE) insulator. The flat-end capacitor was immersed in the sample solution in a glass bottle. This sample section was very suitable for measuring the complex permittivity of strongly polar liquids, such as aqueous solutions, using the TDR system^28^. Dielectric measurements using the TDR technique have the advantage of covering a wide frequency range without full calibration of one port using open, short and load (50 Ω) terminations. However, two waveforms, namely, the reflections from the sample section immersed in a standard material (water in this work) and the sample fluid under the test conditions, are required to obtain the complex permittivity of the sample. Therefore, it is quite difficult to avoid measurement uncertainty, especially regarding the dielectric strength, originating from timing errors due to fluctuations in the sampling time base because it is impossible to record these two reflection waveforms simultaneously. Therefore, in this study, we performed several sets of the waveform measurements (typically six to ten times) and averaged the data to reduce the uncertainty in the measured complex permittivity. In this work, the TDR measurements were performed at 293 K. The final measurement accuracy for the dielectric constant and loss was estimated to be ~±3%. At higher frequencies, between 300 GHz and 2.5 THz, a THz-TDS system was employed. A detailed description has already been published^29^,^30^. The liquid sample cell consisted of a pair of polypropylene windows with 2-mm thickness separated by a metal spacer with 250-μm thickness. The dielectric measurements via the THz-TDS system were performed at 293 K.

## Additional Information

**How to cite this article**: Kawamata, H. *et al*. Hierarchical viscosity of aqueous solution of tilapia scale collagen investigated via dielectric spectroscopy between 500 MHz and 2.5 THz. *Sci. Rep.* **7**, 45398; doi: 10.1038/srep45398 (2017).

**Publisher's note:** Springer Nature remains neutral with regard to jurisdictional claims in published maps and institutional affiliations.

## Figures and Tables

**Table 1:** Viscosity of tilapia collagen aqueous solutions at 293 K.

*c*_collagen_ [wt%]	*η* [mPa · s]
0.0	1
0.4	113
0.8	559
2.0	>1000
4.0	>1000

The value for pure water (1.002 mPa · s) comes from the literature^31^.

**Table 2:** Parameters for analysis of the L process.

(a) amino acid in water								(b) amino acid in collagen aqueous solution	
amino acid	*x*(%)	*τ*_*L*_(ps)	*τ*_*a*_(ps)	a[Å]	b[Å]	*ε*_s_	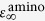	*c*_collagen_(wt%)	*τ*_*a*_ (ps)
glycine	3.0	72	74	3.67	2.11	120	91.3	4.0	74
β-alanine	3.0	107	108	4.30	2.21	135	88.2	4.0	112
L-serine	3.0	123	114	3.93	2.66	123	83.3	0.4	120
L-arginine	1.0	309	282	6.30	2.68	114	74.4	0.4	309

(a) Observed dielectric relaxation time of the L process, *τ*_*L*_, in the aqueous solutions of amino acids and calculated relaxation time, *τ*_*a*_, assuming that the origin of the L process is rotational diffusion of the amino acid molecule. All the parameters used to calculate the values of *τ*_*a*_ with Perrin’s equation (Eq. 2) are also listed in this table. In our calculations, we did not account for the effect of hydrated water surrounding the amino acid molecule because the volume occupied by such water molecules is not well defined. Clearly, the values of observed relaxation times, *τ*_*L*_, for glycine and β-alanine are similar to those of *τ*_*a*_. However, the values of *τ*_*a*_ for L-serine and L-arginine are somewhat less than those obtained experimentally. Because these amino acids have long and charged residuals, it can be assumed that such amino acids are surrounded by a larger amount of hydrated water compared to glycine and β-alanine. For this reason, the experimental relaxation times are greater than the relaxation times estimated using Eq. 2. (b) Collagen concentration, *c*_collagen_, and observed relaxation time of the L process, *τ*_*L*_, in the aqueous solutions of collagen with an amino acid. Note that the values of *τ*_*L*_ in (b) and those in (a) are identical. This fact suggests that the local viscosity of the aqueous solution with collagen is the same as that of pure water.
